# Supplementary material for: Inhibition of NF-κB Signaling by the Reactive Glycolytic Metabolite Methylglyoxal
Source: bioRxiv. 2025 May 16:2025.05.13.653579. Preprint. [Version 1] doi: 10.1101/2025.05.13.653579 (PMC12132462; doi:10.1101/2025.05.13.653579)
Supplement: Supplement 1 [file NIHPP2025.05.13.653579v1-supplement-1.pdf]

bioRxiv preprint doi: https://doi.org/10.1101/2025.05.13.653579; this version posted May 16, 2025. The copyright holder for this preprint (which was not certified by peer review) is the author/funder, who has granted bioRxiv a license to display the preprint in perpetuity. It is made available under aCC-BY 4.0 International license.

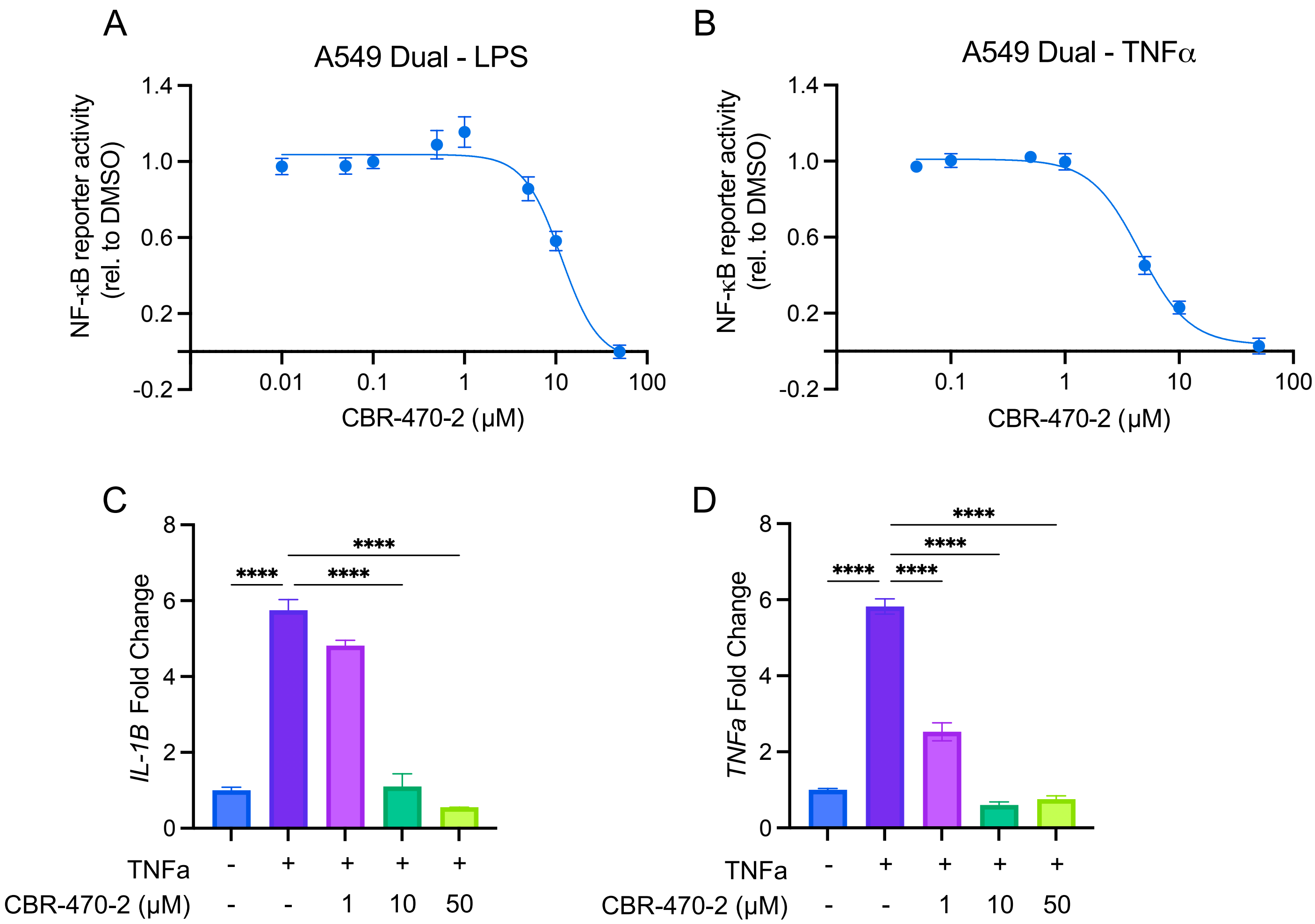

Supplemental Figure 1. CBR-470-2 inhibits NF-κB in multiple cell types and multiple NF-κB stimuli. (A,B) Relative induction of NF-κB as measured by SEAP reporter in A549-Dual cells pretreated with CBR-470-2 for 6 h and then stimulated with LPS (A) or TNF-α (B) overnight. Error bars show SEM for n = 5 replicates (A) or n = 4 replicates (B). (C, D) Relative transcript level of *IL1B* (C) or *TNF* (D) from THP1 cells pretreated with CBR-470-2 for 6 h and then stimulated with TNF-α overnight. Error bars show SEM for n = 3 replicates. ****P<0.0001 for ordinary one-way analysis of variance (ANOVA) with Tukey correction for multiple comparisons between conditions.

bioRxiv preprint doi: https://doi.org/10.1101/2025.05.13.653579; this version posted May 16, 2025. The copyright holder for this preprint (which was not certified by peer review) is the author/funder, who has granted bioRxiv a license to display the preprint in perpetuity. It is made available under aCC-BY 4.0 International license.

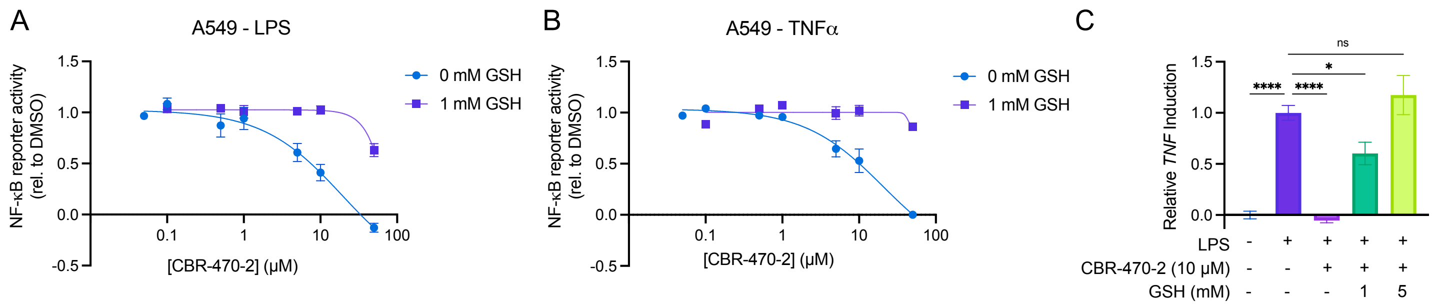

Supplemental Figure 2. CBR-470-2 inhibitory activity is quenched by exogenous GSH. (A, B) Relative induction of NF-κB as measured by SEAP reporter in A549-Dual cells pretreated with 0 or 1 mM GSH for 30 min followed by CBR-470-2 for 6 h and then stimulated with LPS (A) or TNF-α (B) overnight. Error bars show SEM for n = 4 replicates. (C) Relative transcript level of *TNF* from THP1 cells pretreated 0, 1, or 5 mM GSH for 30 min followed by 10 μM CBR-470-2 for 6 h and then stimulated with LPS overnight. Error bars show SEM for n = 3 replicates. *P<0.05, ****P<0.0001 for ordinary one-way analysis of variance (ANOVA) with Dunnett correction for multiple comparisons between conditions.
